# Phosphorylation by Akt within the ST loop of AMPK-α1 down-regulates its activation in tumour cells

**DOI:** 10.1042/BJ20131344

**Published:** 2014-03-28

**Authors:** Simon A. Hawley, Fiona A. Ross, Graeme J. Gowans, Priyanka Tibarewal, Nicholas R. Leslie, D. Grahame Hardie

**Affiliations:** *Division of Cell Signalling & Immunology, College of Life Sciences, University of Dundee, Dundee DD1 5EH, Scotland, U.K.

**Keywords:** Akt, AMP-activated protein kinase (AMPK), cancer, cross-talk, tumour suppressor, ACC, acetyl-CoA carboxylase, AICAR, 5-amino-4-imidazolecarboxamide riboside, AMPK, AMP-activated protein kinase, BRSK, brain-specific kinase, CaMKK, calmodulin-dependent kinase kinase β, DMEM, Dulbecco’s modified Eagle’s medium, GSK3, glycogen synthase kinase 3, HEK, human embryonic kidney, IGF-1, insulin-like growth factor 1, LKB1, liver kinase B1, MEF, mouse embryonic fibroblast, MO25α, mouse protein-25α, mTORC1, mammalian (or mechanistic) target of rapamycin complex 1, NEAA, non-essential amino acid, PI3K, phosphoinositide 3-kinase, PKA, protein kinase A (cAMP-dependent protein kinase), PTEN, phosphatase and tensin homologue deleted on chromosome 10, S6K1, S6 kinase 1, ST loop, serine/threonine-rich loop, STRADα, Ste20-related adapter protein-α, WT, wild-type

## Abstract

The insulin/IGF-1 (insulin-like growth factor 1)-activated protein kinase Akt (also known as protein kinase B) phosphorylates Ser^487^ in the ‘ST loop’ (serine/threonine-rich loop) within the C-terminal domain of AMPK-α1 (AMP-activated protein kinase-α1), leading to inhibition of phosphorylation by upstream kinases at the activating site, Thr^172^. Surprisingly, the equivalent site on AMPK-α2, Ser^491^, is not an Akt target and is modified instead by autophosphorylation. Stimulation of HEK (human embryonic kidney)-293 cells with IGF-1 caused reduced subsequent Thr^172^ phosphorylation and activation of AMPK-α1 in response to the activator A769662 and the Ca^2+^ ionophore A23187, effects we show to be dependent on Akt activation and Ser^487^ phosphorylation. Consistent with this, in three PTEN (phosphatase and tensin homologue deleted on chromosome 10)-null tumour cell lines (in which the lipid phosphatase PTEN that normally restrains the Akt pathway is absent and Akt is thus hyperactivated), AMPK was resistant to activation by A769662. However, full AMPK activation could be restored by pharmacological inhibition of Akt, or by re-expression of active PTEN. We also show that inhibition of Thr^172^ phosphorylation is due to interaction of the phosphorylated ST loop with basic side chains within the αC-helix of the kinase domain. Our findings reveal that a previously unrecognized effect of hyperactivation of Akt in tumour cells is to restrain activation of the LKB1 (liver kinase B1)–AMPK pathway, which would otherwise inhibit cell growth and proliferation.

## Abstract 2

Akt phosphorylates the α1 subunit of AMPK at Ser^487^, leading to reduced Thr^172^ phosphorylation and activation by LKB1. We establish a molecular mechanism for this effect and show that it causes down-regulation of AMPK in several PTEN-null tumour cell lines.

## INTRODUCTION

AMPK (AMP-activated protein kinase) is a ubiquitously expressed sensor of cellular energy status [1], which exists in essentially all eukaryotic cells as heterotrimeric complexes comprising a catalytic α subunit and regulatory β and γ subunits. The α subunits are phosphorylated by upstream kinases at conserved threonine residues within the activation loop (Thr^172^ in rat α1/α2), causing >100-fold activation [2]. Kinases that phosphorylate Thr^172^ include the tumour suppressor LKB1 (liver kinase B1) [3–5], and the Ca^2+^-dependent kinase CaMKKβ (calmodulin-dependent kinase kinase β) [6–8]. The γ subunits contain up to three sites where ADP or AMP bind in competition with ATP [9–12]. Binding of AMP or ADP causes conformational changes that enhance net phosphorylation at Thr^172^ by inhibiting dephosphorylation, whereas binding of AMP, but not ADP, promotes phosphorylation by LKB1 and causes allosteric activation [10,13–15]. The drug A769662 and the natural product salicylate mimic the ability of AMP to inhibit Thr^172^ dephosphorylation and cause allosteric activation, but bind at a different site involving the β subunit [16–18].

AMPK is thus activated by metabolic stresses that deplete ATP, and increase ADP and AMP. Such stresses include compounds inhibiting mitochondrial ATP synthesis, such as the anti-diabetic drugs metformin and phenformin, and the natural products galegine, resveratrol and berberine [19]. Once activated, AMPK switches on catabolic pathways that generate ATP while switching off anabolic pathways and other ATP-requiring processes such as progress through the cell cycle [1]. Thus AMPK has a cytostatic effect on proliferating cells, suggesting that it might exert some of the tumour suppressor effects of its upstream kinase, LKB1. This was supported by recent studies of a mouse B-cell lymphoma model, in which AMPK appeared to act as a negative regulator both of the rapid glycolysis (Warburg effect) and the high growth rate of the tumours [20].

If the LKB1–AMPK pathway acts as a tumour suppressor, one might expect many tumour cells to have been selected for mechanisms that down-regulate it. Indeed, somatic loss-of-function mutations in the gene encoding LKB1 (*STK11*) occur in approximately 30% of non-small-cell lung cancers [21,22] and 20% of cervical cancers [23]. In the HeLa cell, derived from a cervical cancer that had undergone a large deletion in *STK11* [23], increases in AMP and ADP do not enhance Thr^172^ phosphorylation [4] because the basal activity of CaMKKβ is too low to support this unless intracellular Ca^2+^ is also elevated [24]. Somatic mutations in genes encoding AMPK appear to be less frequent in tumours than those in *STK11*, perhaps due to redundancy between multiple AMPK isoforms. However, there may be epigenetic or non-genetic mechanisms by which AMPK is down-regulated in tumour cells. For example, AMPK-α2 expression appears to be frequently down-regulated in hepatocellular carcinoma [25].

The PI3K (phosphoinositide 3-kinase)–Akt (protein kinase B) signalling pathway is frequently activated in tumour cells, often via loss of the tumour suppressor PTEN (phosphatase and tensin homologue deleted on chromosome 10), but also via activating mutations in subunits of PI3K or upstream receptors [26]. Interestingly, Akt phosphorylates the α1 subunit of rat AMPK at Ser^485^ (equivalent to Ser^487^ in human α1), and this was reported to reduce subsequent Thr^172^ phosphorylation and activation by LKB1 [27]. Additionally, PKA (protein kinase A; also known as cAMP-dependent protein kinase) was reported to phosphorylate Ser^485^ with similar effects [28]. High glucose and IGF-1 (insulin-like growth factor 1) also caused phosphorylation of AMPK-α1 at Ser^487^ in porcine vascular smooth muscle cells, and this was associated with reduced Thr^172^ phosphorylation in response to metformin [29]. This mechanism has also been invoked to account for down-regulation of AMPK in human hepatoma cells infected with hepatitis C virus [30]. However, these studies did not provide definitive proof that the Ser^485^/Ser^487^ phosphorylation and the reduced Thr^172^ phosphorylation observed in the intact cells were causally related.

Ser^487^ is located in a loop (residues 472–525 in human α1) that is present in the sequences of α subunits from vertebrates and *Caenorhabditis elegans*, but is truncated or absent in insects (*Drosophila melanogaster*) and fungi (Saccharomyces *cerevisiae*) (Supplementary Figure S1 at http://www.biochemj.org/bj/459/bj4590275add.htm). This loop was disordered in the crystal structure of a partial mammalian αβγ complex expressed in bacteria [11]. We now refer to it as the ‘ST loop’ because it is rich in serine and threonine residues, and it has recently been shown to be phosphorylated by GSK3 (glycogen synthase kinase 3) at Thr^481^ and Ser^477^ (human numbering), which appeared to promote Thr^172^ dephosphorylation [31]. In the present study, we have examined whether phosphorylation of this loop causes down-regulation of AMPK in tumour cells in which the Akt pathway is hyperactivated. We also provide evidence that the ST loop binds to the kinase domain when phosphorylated at Ser^487^ by interactions with the αC helix, thus physically blocking access to Thr^172^.

## EXPERIMENTAL

### Materials and proteins

IGF-1, rapamycin, berberine, phenformin, quercetin and A23187 were from Sigma. MK2206 and A769662 were synthesized by the Division of Signal Transduction Therapy (DSTT) at the University of Dundee. Plasmids expressing the recombinant human α1β2γ1 and α2β2γ1 complexes were gifts from the DSTT and AstraZeneca respectively. Mutations were introduced using the QuikChange® XL site-directed mutagenesis kit (Stratagene). Recombinant human Akt, LKB1 [LKB1—STRADα (Ste20-related adapter protein-α)–MO25α (mouse protein-25α) complex] and BRSK2 (brain-specific kinase 2) were provided by the DSTT.

### Antibodies

Affinity-purified antibodies against AMPK-α subunits [32] and BRSK2 [33], and phospho- and isoform-specific antibodies against Ser^487^ on AMPK-α1 and Ser^491^ on AMPK-α2 [34] were obtained as described previously. Anti-FLAG antibodies were from Sigma and anti-GSK3β antibodies were from Santa Cruz Biotechnology. Phospho-specific antibodies against Thr^172^ on AMPK-α1/α2 (pT172), pan-specific antibodies against Ser^487^/Ser^491^ on AMPK-α1/α2 (pS487/p491), Ser^9^ on GSK3β (pS9), Thr^308^ and Ser^473^ on Akt (pT308, pS473), and Thr^389^ on S6K1 (S6 kinase 1) (pT389) were from Cell Signaling Technology.

### Expression and purification of AMPK in bacteria

Auto-induction medium was inoculated with overnight cultures of LB media containing the appropriate construct. Cultures were grown at 37°C until the *D*_600_ reached ∼0.5, where cultures were placed at 20°C overnight. Bacteria were pelleted by centrifugation (7500 ***g***, 15 min, 4°C), lysed under liquid N_2_ using a pestle and mortar, and resuspended in 50 mM Tris/HCl, pH 8.1, 500 mM NaCl, 20 mM imidazole and EDTA-free protease inhibitor cocktail (Roche). The lysate was purified via the His_6_-tag on the N-terminus of the α subunit using a HisTrap FF column (GE Healthcare Life Sciences). Fractions containing protein were pooled and dialysed into 50 mM sodium/Hepes, pH 8.0, and 200 mM NaCl.

### Sources of cells and cell culture conditions

HEK (human embryonic kidney)-293, DBTRG-05MG, U373-MG and G361 cells were from ECACC (European Collection of Cell Cultures)/HPA (Health Protection Agency) (Porton Down, U.K.) and MDA-MB-468 cells were from A.T.C.C.–LGC Standards. HEK-293 cells stably expressing AMPK-α1/α2 were grown in DMEM (Dulbecco's modified Eagle's medium) containing 4.5 g/l glucose, 10% (v/v) FBS, 100 IU/ml penicillin, 100 μg/ml streptomycin and 200 μg/ml hygromycin. U373-MG cells were grown in MEM (minimal essential medium) containing 10% (v/v) FBS and NEAA (non-essential amino acid) mixture. DBTRG-05 MG cells were grown in RPMI 1640 medium containing 10% (v/v) FBS and NEAA mixture. MDA-MB-468 cells were grown in DMEM containing 4.5 g/l glucose and 10% (v/v) FBS. Lentiviral expression of PTEN or the C124S mutant was as described previously [35]. G361 cells were grown in McCoy's 5A medium containing 10% (v/v) FBS, 100 IU/ml penicillin and 100 μg/ml streptomycin. All cells were switched to serum-free medium containing low (1 g/l) glucose at 16 h before treatment with IGF-1 in the same medium, except that for G361 cells the normal glucose concentration (3 g/l) was maintained.

### AMPK assays in cell-free systems and cell lysates

AMPK activity was measured as described previously [36], but using the AMARA (AMARAASAAALARRR) peptide instead of SAMS (HMRSAMSGLHLVKRR) [37]. Lysates containing stably expressed recombinant FLAG-tagged α subunit were immunoprecipitated from HEK-293 cell lysates (70 μg of protein) by incubation at 4°C for 2 h on a roller mixer with 7 μl of EZview Red anti-FLAG M2 affinity gel (Sigma). After extensive washing, the immunoprecipitates were assayed for AMPK activity as described [38] using the AMARA peptide. Lysates from other cells (which did not express tagged recombinant AMPK) were immunoprecipitated and assayed for AMPK in the same way, except that anti-α1 or anti-α2 antibody bound to Protein G–Sepharose (GE Healthcare) was used in place of anti-FLAG antibody.

### Phosphorylation of GSK3β and AMPK by Akt in cell-free assays

GSK3β (0.5 μg, D200A or D200A/S9A mutants), AMPK (α1β2γ1 complex, D157A or D157A/S487A mutants, or α2β2γ1 complex, D157A or D157A/S491A mutants), were incubated with the indicated amounts of Akt in a final volume of 20 μl for 10 min at 30°C in the presence of 5 mM MgCl_2_ and 200 μM [γ-^32^P]ATP (500 c.p.m./pmol). Incubations were stopped and analysed by autoradiography of membranes after electrophoretic transfer to detect ^32^P incorporation, followed by probing with the indicated antibodies.

### Phosphorylation of AMPK by LKB1 in cell-free assays

AMPK (0.5 μg, with or without prior phosphorylation by Akt on Ser^487^) was incubated with the amount of LKB1 indicated in the Figure legends in a final volume of 20 μl for 10 min at 30°C in the presence of 5 mM MgCl_2_ and 200 μM ATP. AMPK activity was subsequently determined by transferring 5 μl from this reaction mixture to an AMPK assay, as described below. The remaining 15 μl was retained for analysis by Western blotting.

### Generation of HEK-293 cells stably expressing α1, α2 or specified mutations

DNAs encoding full-length human α1 and α2 were amplified with primers designed to encode a 5′ KpnI site, and a 3′ FLAG-tag followed by an XhoI site. The resulting PCR products were cloned into the pcDNA5/FRT plasmid (Invitrogen). Stable cell lines were generated and cultured as described previously [19].

### Incubation of HEK-293 cells with IGF-1 and various activators and inhibitors

HEK-293 cells, stably expressing AMPK [α1 WT (wild-type), α1-S487A or α2 WT as indicated] were grown to ≈80% confluence and then serum-starved for 16 h. Cells were then treated as described in the Figure legends. Pre-treatments with MK2206 (3 μM) were for 30 min. Pre-treatments with rapamycin (100 nM) were for 45 min. Incubations in the presence of 30 ng/ml IGF-1 were for 20 min. Treatments with A769662 (300 μM) were for 40 min (unless otherwise indicated) and those with berberine (300 μM) for 60 min.

### Cloning, expression, purification and phosphorylation of the ST loop peptide

DNA encoding residues 466–525 from human AMPK-α1 were amplified by PCR to include an N-terminal XhoI site and a C-terminal His_6_-tag followed by a KpnI site, allowing insertion into pGEXKG (GE Healthcare Life Sciences). Cultures were grown at 37°C until the *D*_600_ reached ∼0.6, when cultures were induced with 1 mM IPTG and kept at 20°C overnight. The bacteria were pelleted by centrifugation (7500 ***g***, 15 min, 4°C), lysed under liquid N_2_ using a pestle and mortar, and resuspended in 50 mM Tris/HCl, pH 8.1, 500 mM NaCl, 20 mM imidazole and the EDTA-free protease inhibitor cocktail (Roche). The protein was purified using a HisTrap FF column (GE Healthcare Life Sciences). Fractions containing protein were pooled and incubated for 30 min at 30°C with 5 mM MgCl_2_ and 200 μM ATP-γ-phosphorothioate in the presence or absence of His_6_-tagged Akt. The mixture was then applied to a 1 ml GST FF column (GE Healthcare Life Sciences). After washing, the column was loaded with thrombin protease (Sigma) in 50 mM sodium/Hepes, pH 8, and 200 mM NaCl, capped, and left overnight at 4°C. Flow-through fractions containing cleaved phosphorylated or non-phosphorylated peptide was collected.

### Western blotting and other analytical procedures

For analysis of ACC (acetyl-CoA carboxylase) phosphorylation, SDS/PAGE was performed using precast Novex NuPAGE Tris-Acetate 3–8% gradient polyacrylamide gels in the Tris-Acetate SDS buffer system. For analysis of all other proteins, SDS/PAGE was performed using precast Novex NuPAGE Bis-Tris 4–12% gradient polyacrylamide gels in the Mops buffer system (Invitrogen). Proteins were transferred to nitrocellulose membranes (Bio-Rad Laboratories) using the Xcell blot module. Membranes were blocked in LI-COR Odyssey blocking buffer for 1 h, and detection performed using the appropriate secondary antibody coupled with IR680 or IR800 dye. Membranes were scanned using the LI-COR Odyssey IR imager.

### Statistical analysis

Unless stated otherwise, statistical significance was tested using GraphPad Prism 5 by one-way ANOVA, with Bonferroni's multiple comparison tests of the selected datasets as shown in the Figures.

## RESULTS

### Akt phosphorylates Ser^487^ on AMPK-α1, but not the equivalent site on AMPK-α2

**Figure 1:**
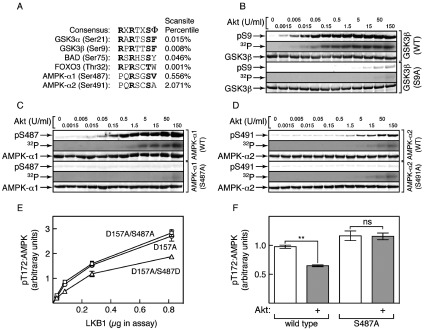
Phosphorylation by Akt of inactive α1 at Ser^487^, but not α2 at Ser^491^, inhibits Thr^172^ phosphorylation by LKB1 (**A**) Alignment of sequences around Ser^487^ on α1 and Ser^491^ on α2 with the consensus for Akt phosphorylation [39] and with sequences around established Akt target sites. The right-hand column shows the percentile score for the sequence as a potential Akt target computed using Scansite (http://scansite.mit.edu/). (**B**) Phosphorylation of GSK3β by the indicated amounts of Akt. Incubations were analysed by Western blotting with the indicated antibodies, or by autoradiography to detect ^32^P radioactivity. (**C**) As with (**B**), but analysing phosphorylation of AMPK (α1β2γ1 complex). (**D**) As with (**B**), but analysing phosphorylation of AMPK (α2β2γ1 complex). (**E**) Rate of phosphorylation by LKB1 (signal using an anti-pT172 antibody expressed as a ratio of signal using an anti-AMPK-α antibody) obtained using the indicated mutants of the α1β2γ1 complex. Results are means±S.E.M. (*n*=2). (**F**) Rate of phosphorylation by LKB1 (signal obtained using an anti-pT172 antibody expressed as a ratio of signal using an anti-AMPK-α antibody) following pre-incubation with MgATP with or without Akt (30 units/ml, 20 min), for the inactive (D157A) α1β2γ1 complex or an S487A/D157A double mutant. Results are means±S.E.M. (*n*=4); **P<0.01; ns, not significant.

Akt phosphorylates serine or threonine residues within the sequence motif RXRXXS/TΦ, where Φ is a bulky hydrophobic residue [39]. Figure 1(A) shows an alignment of this consensus with sequences around some established Akt targets, the ST loop sequence containing Ser^487^ in human α1, and the equivalent site on α2 (Ser^491^). Both of the latter have a serine residue at P-2, with Akt having a preference for the serine or threonine residue at this position [39]. However, both also have a proline residue rather than arginine at the P-5 position, and Ser^491^ also has an alanine residue rather than a bulky hydrophobic residue at P+1. Neither is therefore a perfect fit to the Akt consensus; Scansite 2.0 [40] identifies Ser^487^ as a potential Akt site only at medium stringency, and Ser^491^ only at low stringency.

To avoid complications caused by autophosphorylation, we initially used inactive mutants of human AMPK complexes (α1-D159A/β2/γ1 and α2-D157A/β2/γ1), with or without α1-S487A or α2-S491A mutations, as substrates for phosphorylation by human Akt. For comparison, we used an inactive (D200A) mutant of the known Akt substrate GSK3β, with or without mutation of the Akt site (Ser^9^). Interestingly, AMPK-α1 was a reasonable substrate for Akt although not as good as GSK3, whereas AMPK-α2 was a very poor substrate. Using either an anti-pS9 antibody or by ^32^P-labelling, phosphorylation of GSK3β was saturated at 0.5 unit/ml Akt, when the phosphorylation stoichiometry by ^32^P-labelling was 1.03 mol/mol (Figure 1B). The phosphorylation of AMPK-α1 within the α1β2γ1 complex was only saturated at 5 units/ml, when the stoichiometry was 0.96 mol/mol (Figure 1C). For both GSK3β and AMPK-α1, the signals obtained using phospho-specific antibodies (pS9/pS487) or ^32^P-labelling were abolished by mutation of the respective sites to alanine (S9A/S487A). By contrast, there was much less phosphorylation of Ser^491^ within the α2β2γ1 complex (Figure 1D). By ^32^P-labelling, the stoichiometry of α2 phosphorylation was only 0.18 mol/mol, even with Akt at 30- and 300-fold higher concentrations than those required to obtain stoichiometric phosphorylation of α1 and GSK3β respectively. Although we did detect some phosphorylation of Ser^491^ using a phospho-specific antibody and this was abolished in a S491A mutant, ^32^P-labelling was not affected by the S491A mutation, suggesting that the low level of α2 phosphorylation by Akt was mainly accounted for by modification at other site(s).

### Ser^487^ phosphorylation reduces Thr^172^ phosphorylation: studies with inactive AMPK

We next tested the ability of the human LKB1–STRADα–MO25α complex to phosphorylate Thr^172^ in an inactive (α1-D157A) human α1β2γ1 complex. Before phosphorylation by Akt, the rate of phosphorylation of Thr^172^ by the LKB1 complex was unaffected by an S487A mutation, although an S487D mutation reduced the rate of Thr^172^ phosphorylation by approximately 30% (Figure 1E). When the inactive (D157A) mutant complex was first phosphorylated by Akt under conditions that yielded stoichiometric Ser^487^ phosphorylation, subsequent phosphorylation of Thr^172^ was reduced by approximately 40%, an effect abolished by an S487A mutation (Figure 1F).

### Phosphorylation of Ser^491^ on AMPK-α2 is due to autophosphorylation

**Figure 2:**
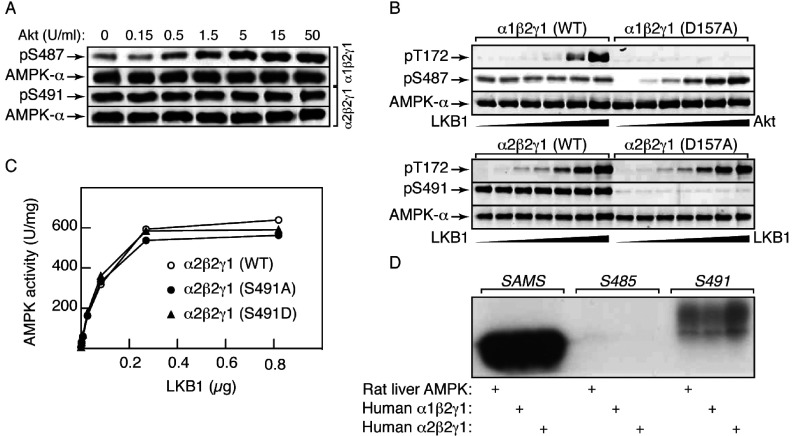
Ser^491^ (AMPK-α2), and to a lesser extent Ser^487^ (AMPK-α1), are caused by autophosphorylation (**A**) AMPK (α1β2γ1 or α2β2γ1 complex) was incubated with MgATP and the indicated concentrations of Akt for 20 min at 30°C, and aliquots (20 μl) analysed by Western blotting using the indicated antibodies. (**B**) AMPK (α1β2γ1 or α2β2γ1, WT or inactive D157A mutant) was incubated with MgATP and increasing concentrations of LKB1 (0, 8.2, 28, 82, 280 and 820 ng) or Akt (0, 0.15, 0.5, 1.5, 5 and 15 units/ml) for 10 min at 30°C. (**C**) AMPK (α2β2γ1 complex, WT, S491A or S491D mutant) was incubated with MgATP and the indicated amount of LKB1 in a final volume of 20 μl for 10 min at 30°C, and AMPK activity determined. (**D**) Phosphorylation of synthetic peptides by three different preparations of AMPK. AMPK (0.1 unit, either α1β2γ1, α2β2γ1 complexes or purified from rat liver [2]) was incubated with 5 nmol of the SAMS, S485 or S491 peptides in the presence of 5 mM MgCl_2_ and 200 μM [γ-^32^P]ATP in a final volume of 25 μl for 30 min at 30°C, before SDS/PAGE and autoradiography.

We next tested the effects of Akt on either WT, S487A or S491A mutants of active α1β2γ1 and α2β2γ1 complexes. Figure 2(A) shows that with the α1β2γ1 complex there was slight phosphorylation of Ser^487^ even in the absence of Akt, although phosphorylation of this site increased markedly with increasing Akt. By contrast, Ser^491^ in an α2β2γ1 complex appeared to be fully phosphorylated in the absence of Akt, and addition of Akt had no further effect. These results suggested that Ser^491^ was modified by autophosphorylation, whereas Ser^487^ is phosphorylated by Akt, with a small degree of autophosphorylation. Consistent with this, there was substantial phosphorylation of Ser^491^ in a human α2β2γ1 complex, and slight phosphorylation of Ser^487^ in a human α1β2γ1 complex, when they were incubated with MgATP alone; these effects were completely abolished by D157A mutations that rendered the complexes inactive, although Ser^487^ could still be phosphorylated by Akt in the inactive complex (Figure 2B). Unlike the equivalent α1β2γ1 complexes, where the S487D mutant was phosphorylated at a lower rate (Figure 1E), the WT, S491A and S491D mutant α2β2γ1 complexes were phosphorylated on Thr^172^ at equal rates by LKB1 (Figure 2C).

We also synthesized peptides corresponding to the sequences around Ser^485^ on rat α1 (S485, TPQRSGSISNYRS) or Ser^491^ on rat/human α2 (S491, TPQRSCSAAGLHR), and compared them as AMPK substrates with the classical SAMS peptide [36]. Although the SAMS peptide was by far the best substrate, the S491 peptide was also phosphorylated, whereas the S485 peptide was not phosphorylated at all. The results were identical with rat liver AMPK (a mixture of α1β1γ1 and α2β1γ1 complexes) or with recombinant human α1β2γ1 and α2β2γ1 complexes (Figure 2D).

### Ser^487^ phosphorylation reduces Thr^172^ phosphorylation: studies with active AMPK

**Figure 3:**
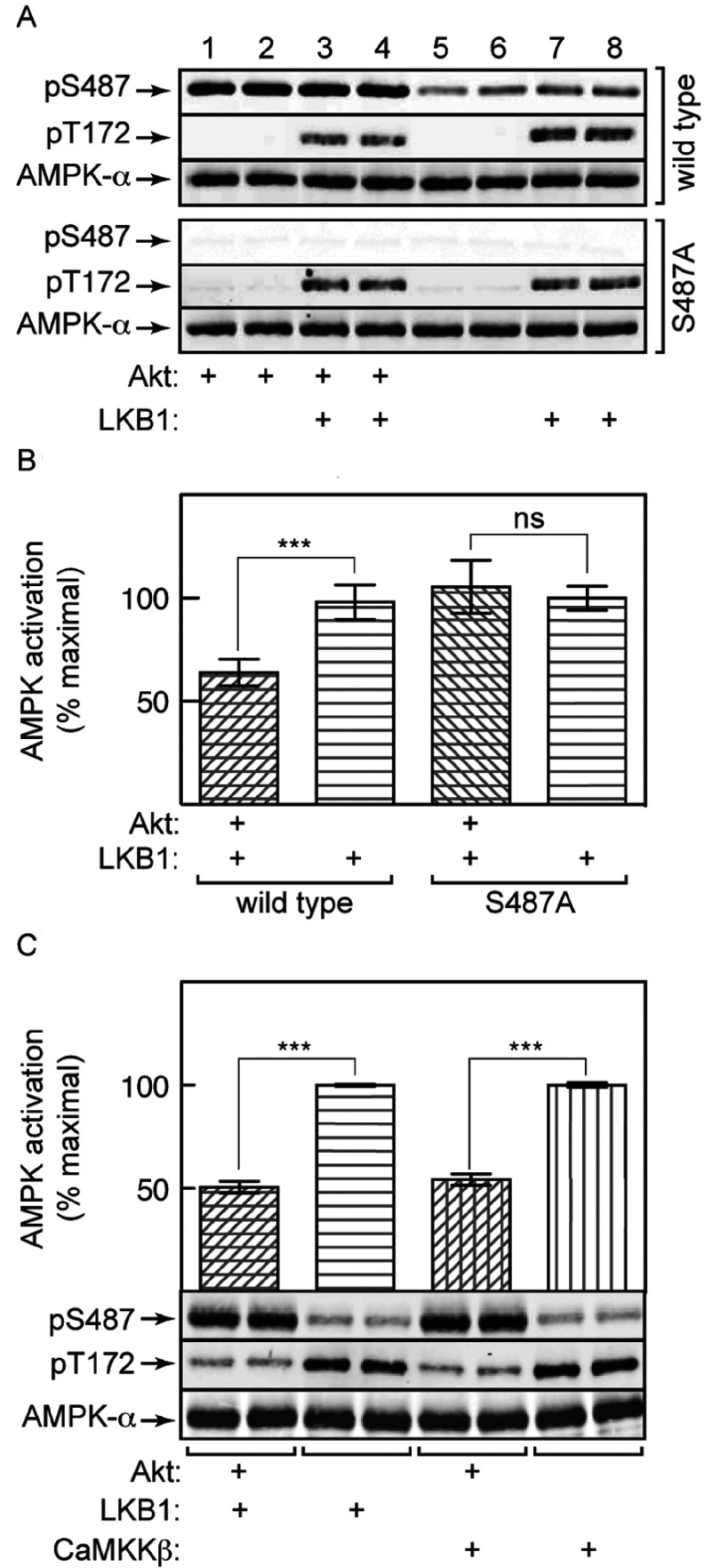
Prior phosphorylation of AMPK-α1 by Akt at Ser^487^ reduces subsequent phosphorylation and activation of Thr^172^ by LKB1 or CaMKKβ AMPK (α1β2γ1 complex, WT or S487A mutant) was pre-incubated with MgATP and Akt for 30 min, and then with LKB1 (50 ng) or CaMKKβ (23 ng) for 10 min at 30°C in a final volume of 40 μl. (**A**) Phosphorylation of AMPK by LKB1 in the absence or presence of Akt. (**B**) Activation of AMPK by LKB1 in the absence or presence of Akt. (**C**) Activation/phosphorylation by LKB1/CaMKKβ in the absence or presence of Akt. Phosphorylation (**A** and **C**) was assessed in duplicate samples by Western blotting, and AMPK activity (**B** and **C**) by kinase assays. Results in (**B**) and (**C**) are means±S.E.M. (*n*=4); ***P<0.001; ns, not significant.

We next incubated the active α1β2γ1 complex with Akt under conditions where we obtained stoichiometric phosphorylation of Ser^487^, and subsequently treated with the LKB1 complex under conditions where we could measure the initial rate of Thr^172^ phosphorylation and consequent activation. As with the inactive complex (Figure 1F), prior Akt phosphorylation reduced the rate of subsequent Thr^172^ phosphorylation (Figure 3A), but using the active complex this could also be seen to be associated with a reduction in activation by LKB1 of approximately 40% (Figure 3B); both effects were abolished by an S487A mutation within the ST loop. Figure 3(C) shows that the inhibitory effect of prior Ser^487^ phosphorylation on Thr^172^ phosphorylation and AMPK activation was identical using either LKB1 or CaMKKβ, showing that the effect is independent of the upstream kinase utilized.

### Phosphorylation of Ser^487^ in intact cells reduces LKB1-dependent AMPK activation

**Figure 4:**
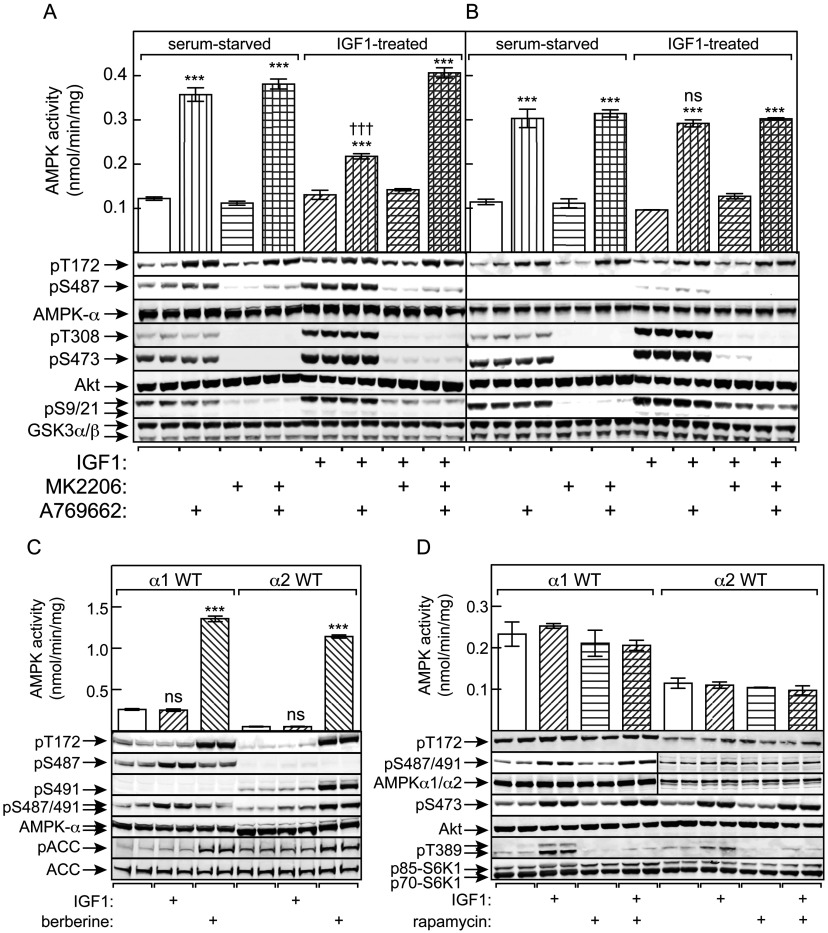
Phosphorylation of Ser^487^ on AMPK-α1 by Akt in HEK-293 cells inhibits subsequent phosphorylation of Thr^172^ and AMPK activation, Ser^491^ on AMPK-α2 is modified by autophosphorylation, and Ser^487^ phosphorylation is rapamycin-insensitive HEK-293 cells stably expressing WT AMPK (**A**) or an S487A mutant (**B**) were serum-starved overnight and then incubated with IGF-1 in the presence or absence of MK2206 as described in the Experimental section. The cells were then treated with or without A769662 (300 μM for 40 min) and lysates prepared for immunoprecipitate kinase assay and Western blots. Blots are samples from separate dishes (*n*=2), whereas activity data are means±S.E.M. (*n*=4); ***P<0.001 compared with relevant control without A769662; †††*P*<0.001; ns, not significant, for IGF-1-treated against relevant serum-starved control. (**C**) Cells expressing WT AMPK-α1 or AMPK-α2 were treated with IGF-1 (30 ng/ml) or berberine (300 μM) and lysates analysed by immunoprecipitate kinase assays and Western blotting (two separate dishes). Activity data are means±S.E.M. (*n*=4); ***P<0.001; ns, not significant, compared with control without IGF-1 or berberine. (**D**) Cells expressing WT AMPK-α1 or AMPK-α2 were treated with IGF-1 in the absence or presence of rapamycin (100 μM) and lysates analysed by immunoprecipitate kinase assays and Western blotting. Activity data are means±S.E.M. (*n*=2); duplicate blots were from separate dishes.

To test the effects of Ser^487^ phosphorylation in intact cells, we generated isogenic HEK-293 cells stably expressing FLAG-tagged WT AMPK-α1 or AMPK-α2, or a non-phosphorylatable (S487A) α1 mutant. We have shown previously that when AMPK-β [16] or AMPK-γ [19] subunits are expressed using this system, they largely replace the endogenous subunit because they compete for the available α/γ or α/β partners, with free subunits being unstable. This was also true in the present study because we showed that approximately 70% of the total AMPK activity could be immunoprecipitated using anti-FLAG antibody, with the remaining 30% (representing a small proportion with endogenous α subunits) being subsequently precipitated using anti-α1 or anti-α2 antibodies. The presence of a small amount of endogenous α subunits does not affect interpretation of the kinase assays shown in Figure 4, which were conducted in anti-FLAG antibody immunoprecipitates, but a small proportion of AMPK-α subunits detected in the Western blots (e.g. the faint signal obtained using the anti-pS487 antibody in the cells expressing the S487A mutant) may be due to these endogenous subunits.

The cells expressing AMPK-α1 were serum-starved, and some were then treated with IGF-1 to activate Akt. As expected, IGF-1 resulted in marked increases in phosphorylation of the activating sites on Akt (Thr^308^ and Ser^473^), which were blocked by the Akt inhibitor MK2206 [41], as was the phosphorylation of Ser^9^/Ser^21^ on GSK3-α/β (markers of Akt activation) and Ser^487^ on AMPK-α1 (Figure 4A). When cells expressing WT α1 were treated with A769662, the activation of AMPK, and Thr^172^ phosphorylation, was markedly blunted if the cells had been exposed previously to IGF-1, an effect abolished by MK2206. The effect of IGF-1 to reduce AMPK activation and phosphorylation of Thr^172^ correlated with increased Ser^487^ phosphorylation, and was absent in cells expressing the S487A mutant (Figure 4B).

### Ser^491^ on AMPK-α2 is not phosphorylated by Akt, but by autophosphorylation

Figure 4(C) shows results obtained when serum-starved cells expressing WT AMPK-α1 or AMPK-α2 were treated either with IGF-1 or with berberine, which activates AMPK by inhibiting mitochondrial ATP synthesis [19]. As expected, treatment of α1-expressing cells with IGF-1 caused increased phosphorylation of Ser^487^, but not Thr^172^, whereas treatment with berberine caused increased phosphorylation of Thr^172^, accompanied by AMPK activation, but not Ser^487^. This contrasted markedly with results in α2-expressing cells, where treatment with IGF-1 did not increase phosphorylation of Ser^491^, whereas treatment with berberine caused increased phosphorylation of both Ser^491^ and Thr^172^, together with AMPK activation. The results for phosphorylation of Ser^487^ and Ser^491^ were very similar whether we used in-house phospho-specific antibodies that are isoform-specific, or a commercial antibody that recognizes the equivalent sites on both α1 and α2. These results are consistent with the results in Figures 2 and 3, showing that Ser^491^ on α2 is modified by autophosphorylation, and not by Akt as for Ser^487^ on α1. As expected, increased phosphorylation of the downstream AMPK target ACC correlated with Thr^172^ phosphorylation and AMPK activation in both cell lines.

### Ser^487^ is phosphorylated by Akt and not by a kinase downstream of mTORC1 [mammalian (or mechanistic) target of rapamycin complex 1]

To confirm that Ser^487^ was phosphorylated directly by Akt in the cells, and not by a downstream kinase such as p70 S6K1, we tested the effects of rapamycin, an inhibitor of mTORC1. Rapamycin did not block the IGF-1-stimulated phosphorylation of Ser^487^ on α1 or Ser^473^ on Akt although, as expected, it blocked phosphorylation of an established mTORC1 substrate, Thr^389^ on the p70/p85 isoforms of S6K1 (Figure 4D).

### Phosphorylation of Ser^487^ in intact cells reduces CaMKKβ-dependent AMPK activation

**Figure 5:**
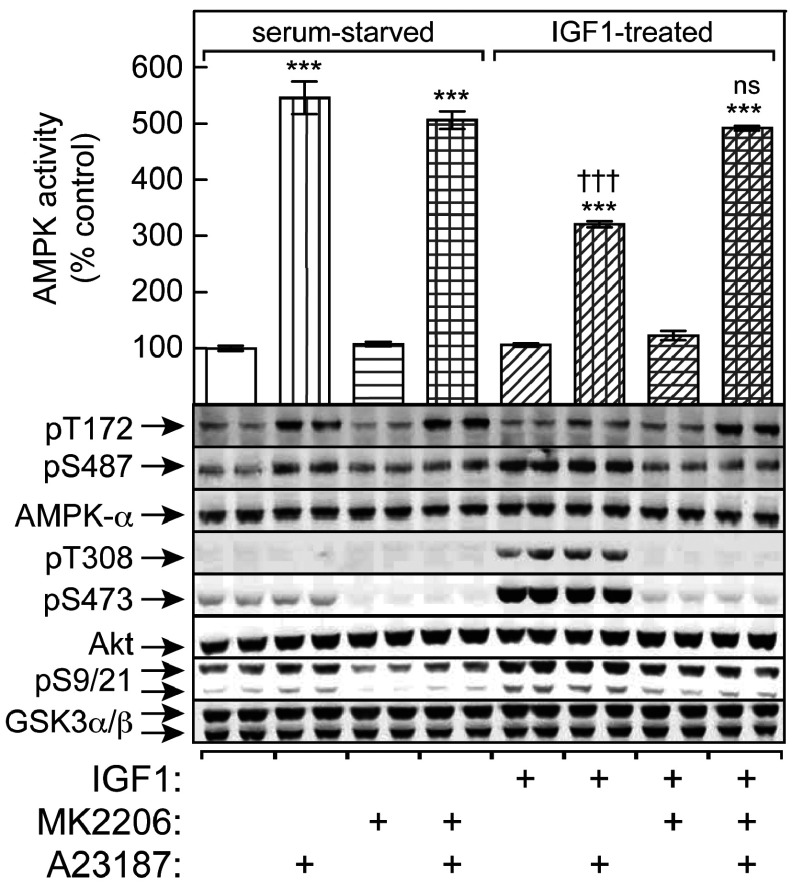
Phosphorylation of Ser^487^ on AMPK-α1 by Akt in G361 cells inhibits subsequent phosphorylation of Thr^172^ and AMPK activation by CaMKKβ G361 cells were serum-starved overnight and then incubated with IGF-1 in the presence or absence of MK2206 as described in the Experimental section. Cells were then treated with or without A23817 (30 nM for 30 min) and lysates prepared for immunoprecipitate kinase assay and Western blots. Blots are samples from separate dishes, whereas activity data are means±S.E.M. (*n*=4); ***P<0.001 compared with the relevant control without A23187; †††*P*<0.001; ns, not significant, for IGF-1-treated against relevant serum-starved control.

To show that phosphorylation of Ser^487^ on AMPK-α1 by Akt could also reduce subsequent activation by CaMKKβ, we used the LKB1-null G361 melanoma cell line. The cells were serum-starved, and some were then treated with IGF-1 to activate Akt. As expected, IGF-1 resulted in marked increases in phosphorylation of Thr^308^ and Ser^473^ on Akt, Ser^9^/Ser^21^ on GSK3α/β and Ser^487^ on AMPK-α1, all of which were blocked or reduced by MK2206 (Figure 5). When the cells were treated with the Ca^2+^ ionophore A23187 to activate CaMKKβ, the activation of AMPK and Thr^172^ phosphorylation, was significantly blunted if the cells had been exposed previously to IGF-1, an effect completely abolished by MK2206 (Figure 5).

### AMPK activation is reduced in PTEN-null tumour cells in an Akt-dependent manner

To examine whether hyperactivation of Akt in tumour cells due to PTEN loss might also inhibit AMPK activation, we initially examined the PTEN-null glioblastoma cell line DBTRG-05MG. We first tested a range of AMPK activators to see whether any interfered with Akt activation. Surprisingly, phenformin and quercetin blocked phosphorylation of Akt at Ser^473^, although berberine, A769662 and A23187 did not (Supplementary Figure S2A at http://www.biochemj.org/bj/459/bj4590275add.htm). As expected, all agents also increased the phosphorylation of ACC1 at Ser^79^ and AMPK at Thr^172^ in MEFs (mouse embryonic fibroblasts), although the effect of A769662 on Thr^172^ phosphorylation was small, indicating that it was mainly acting through an allosteric mechanism (Supplementary Figure S2B). The inhibitory effects of phenformin and quercetin on Akt Ser^473^ phosphorylation were observed in WT MEFs, but were ‘off-target’ AMPK-independent effects, because they were also observed in double-knockout (α1^−/−^ α2^−/−^) MEFs (Supplementary Figure S2B and S2C). To avoid this complication, in subsequent studies we focused on the effects of A769662, which activates AMPK by direct binding to the β subunit [16,17] and does not inhibit ATP synthesis [19] or Akt Ser^473^ phosphorylation (Supplementary Figure S2).

**Figure 6:**
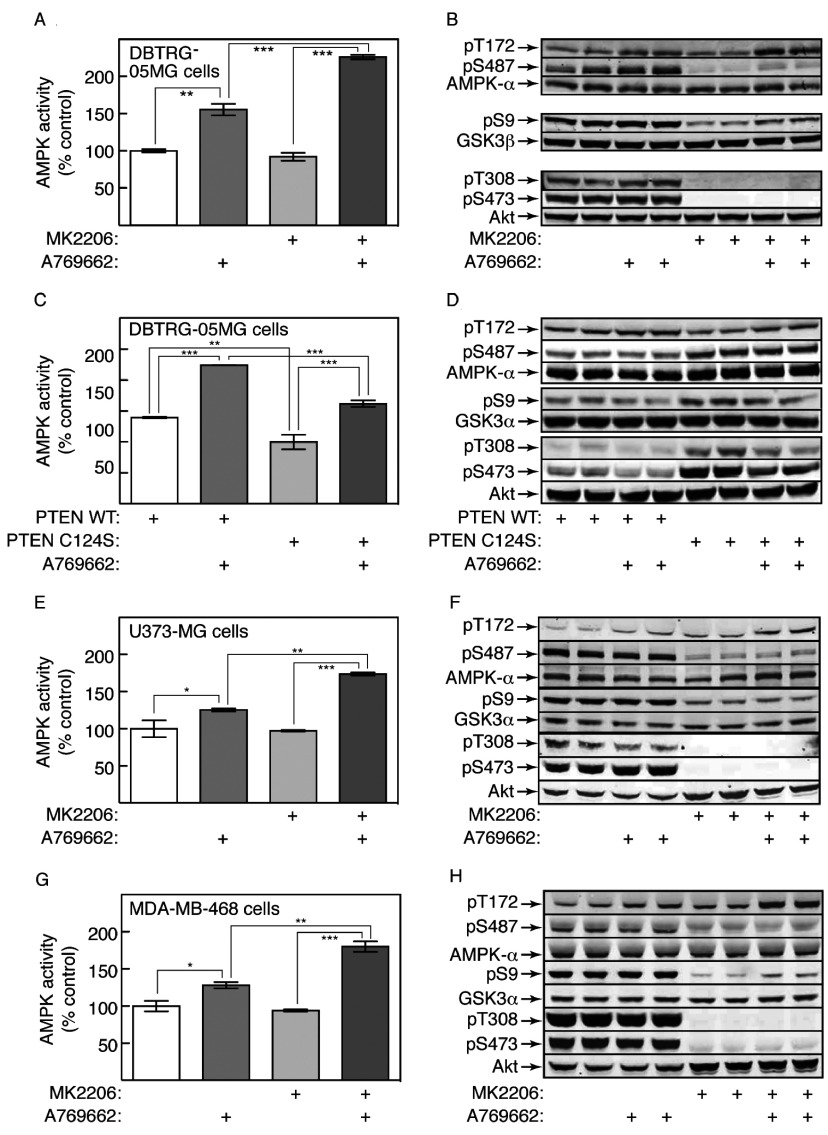
Prior IGF-1 treatment down-regulates AMPK activation by A769662 in PTEN-null tumour cell lines (**A**) Glioblastoma (DBTRG-05MG) cells were incubated for 20 min with or without MK2206 (3 μM), followed by 60 min with or without A769662 (300 μM). Lysates were then analysed for AMPK activity. Results are means±S.E.M. (*n*=2); ***P<0.001; **P<0.01. (**B**) Analysis of the samples from (**A**) by Western blotting using the indicated antibodies (duplicate dishes). (**C**) WT PTEN or a phosphatase-inactive (C124S) mutant were expressed in DBTRG-05MG cells using lentiviral vectors, and the effects of A769662 tested as in (**A**). Results are means±S.E.M. (*n*=2 for WT PTEN, *n*=4 for C124S PTEN); ***P<0.001; **P<0.01. (**D**) Analysis of the samples from (**C**) by Western blotting using the antibodies shown (duplicate dishes). (**E** and **F**) Glioblastoma (U373-MG) cells were incubated and analysed as in (**A** and **B**). (**G** and **H**) Breast cancer (MDA-MB-468) cells were incubated and analysed as in (**A** and **B**). For (**E**) and (**G**), results are means±S.E.M. (*n*=2); ***P<0.001; **P<0.01; *P<0.05.

AMPK in DBTRG-05MG cells was activated by A769662, but activation (Figure 6A) and Thr^172^ phosphorylation (Figure 6B) were greatly enhanced when the selective Akt inhibitor MK2206 was added before A769662. Thus reduced activation of AMPK in these cells was Akt-dependent. As expected, inhibition of Akt by MK2206 was associated with greatly reduced phosphorylation of Ser^487^, and of the Akt site on GSK3β, Ser^9^ (Figure 6B). As reported previously [41], MK2206 also blocked the phosphorylation of Akt at the activating sites, Thr^308^ and Ser^473^.

We also examined the effect of re-expressing PTEN in DBTRG-05MG cells, using a lentiviral vector that gives levels of expression similar to normal cells. As a control, we expressed a C124S mutant that has no lipid phosphatase activity. Prior expression of WT PTEN enhanced the activation (Figure 6C) and Thr^172^ phosphorylation (Figure 6D) of AMPK in response to A769662. When compared with the C124S mutant, expression of WT PTEN was associated with decreased phosphorylation of Ser^487^ on AMPK-α1 and Ser^9^ on GSK3β, and markedly decreased phosphorylation of Thr^308^ and Ser^473^ on Akt (Figure 6D).

We also studied two other PTEN-null human cell lines, i.e. U373-MG (another glioblastoma line) and MDA-MB-468 (a breast cancer line). Similar to the DBTRG-05MG cells, there was a modest activation (Figures 6E and 6G) and Thr^172^ phosphorylation (Figures 6F and 6H) of AMPK in response to A769662, but both were enhanced when Akt was inhibited using MK2206.

### The phosphorylated ST loop interacts with the kinase domain, hindering access to Thr^172^

**Figure 7:**
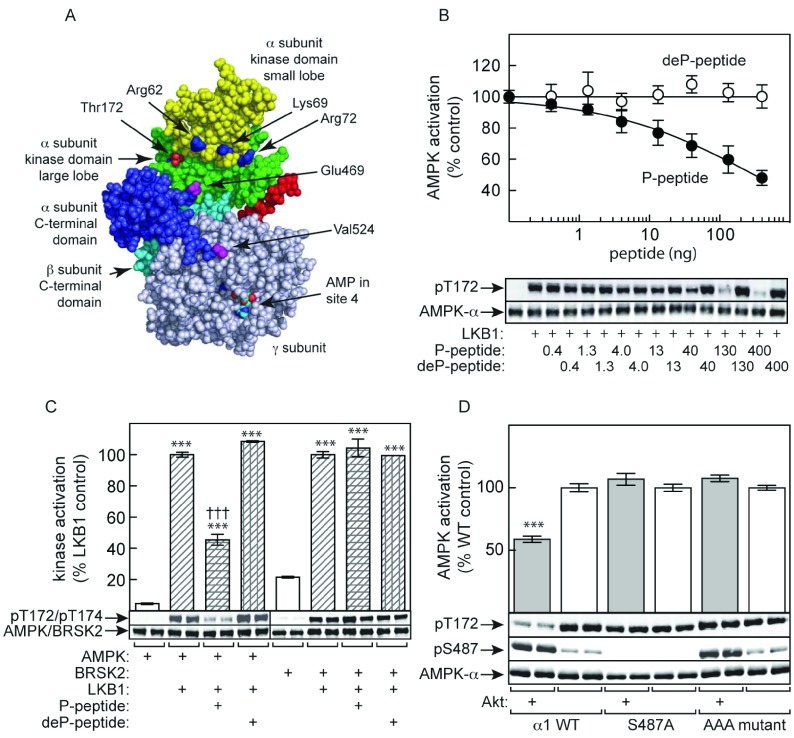
Evidence that the phosphorylated ST loop inhibits LKB1 phosphorylation by direct interaction with the kinase domain (**A**) Model for the structure of an AMPK heterotrimer (PDB code 2Y8L, space-filling model made using PyMOL; http:://pymol.org) showing the location of the ends of the ST loop (Glu^469^ and Val^524^), with Glu^469^ particularly close to Thr^172^; the intervening residues of the ST loop were deleted from the construct used to produce this structure. (**B**) Inhibition of activation of the human α1β2γ1 complex by LKB1 by peptide corresponding to the sequence from Arg^466^ to Asp^525^, either with (P-peptide) or without (deP-peptide) prior phosphorylation by Akt. Results are means±S.E.M. (*n*=4). (**C**) Inhibition of activation and phosphorylation by LKB1 of α1β2γ1 complex (left-hand side) and BRSK2 (right-hand side) by P-peptide and deP-peptide as in (**B**). Results for kinase activity are expressed as a percentage of activities obtained without either peptide, and are means±S.E.M. (*n*=2); ***P<0.001 relative to controls without LKB1; †††*P*<0.001 relative to control without P-peptide. Results of Western blots are from duplicate incubations. (**D**) Human AMPK (α1β2γ1 complex, either WT, S487A or AAA mutant) was incubated with LKB1 following prior incubation with ATP with or without Akt. AMPK activation (top panel) and phosphorylation of Thr^172^ and Ser^487^ (bottom panel) was monitored.

We next addressed the mechanism by which Ser^487^ phosphorylation inhibits subsequent Thr^172^ phosphorylation. In the structure of a partial mammalian α1β2γ1 complex, the ST loop from Ile^470^ to Asp^523^ (rat numbering) was disordered [11]; the complex had been expressed in bacteria, so the ST loop was likely to be unphosphorylated. In a subsequent structure [10], the ST loop was deleted as it was thought that it might hinder crystallization. However, the location of the residues at the ends of the loop (Glu^469^ and Val^524^) in this structure show that they lie close to the kinase domain, being approximately 20 and 40 Å (1 Å=0.1 nm) from Thr^172^ respectively (Figure 7A). We hypothesized that the ST loop might interact with the kinase domain when phosphorylated on Ser^487^. We also noticed three basic residues, Arg^62^, Lys^69^ and Arg^72^ (rat α1 numbering), which are located within the ‘αC helix’ of the small lobe of the kinase domain. An extension of our hypothesis was that phosphate groups on the ST loop interact with these basic side chains, triggering a stable interaction between the ST loop and the kinase domain that physically blocks access of Thr^172^ to upstream kinases.

To test the first hypothesis, we made a construct expressing the peptide from Arg^466^ to Asp^525^ on human α1 as a GST fusion. The protein was expressed in and purified from bacteria, the GST domain removed, and the remaining peptide (Arg^466^–Asp^525^) either thiophosphorylated using ATPγS (adenosine 5′-[γ-thio]triphosphate) and Akt (thiophosphate being resistant to protein phosphatases), or left unphosphorylated. Consistent with our hypothesis, the phosphopeptide caused a concentration-dependent inhibition of activation and Thr^172^ phosphorylation of the bacterially expressed α1β2γ1 complex by LKB1, although we were unable to generate the peptide at high enough concentrations to fully saturate inhibition. By contrast, the unphosphorylated peptide had no effect (Figure 7B). To confirm that the inhibition of activation loop phosphorylation was specific to AMPK, we showed that neither the phospho- nor the dephospho-peptide inhibited activation and phosphorylation of the AMPK-related kinase BRSK2 by LKB1 (Figure 7C).

To test the extended hypothesis, we made a triple mutation (R64A/K71A/R74A, human α1 numbering, ‘AAA mutant’), in the context of the recombinant human α1β2γ1 complex used in Figure 3, of the three basic residues in the kinase domain that we propose to interact with phosphate groups on the ST loop. Consistent with our hypothesis, prior phosphorylation of the WT complex by Akt reduced subsequent activation and Thr^172^ phosphorylation by LKB1, but this effect was completely abolished by either an S487A mutant or an AAA mutant, even though the latter was still phosphorylated on Ser^487^ by Akt (Figure 7D).

## DISCUSSION

It has been reported previously that prior phosphorylation of Ser^485^ on rat AMPK-α1 by Akt, within a rat α1β1γ1 complex, caused a 40% reduction in the rate of subsequent phosphorylation of Thr^172^ and activation by LKB1 in cell-free assays, an effect that was abolished by a non-phosphorylatable S485A substitution [27]. In the present study, we have followed up this observation and made several new findings. First, we demonstrated identical effects with the equivalent site (Ser^487^) on the human α1β2γ1 complex (Figures 1F and 4), showing that the effect is conserved in humans and is also independent of the β subunit isoform. Secondly, we show that the effect is not specific to the upstream kinase LKB1, but is also observed with CaMKKβ (Figure 3C). Thirdly, we report that Ser^491^ (the site equivalent to Ser^487^ in AMPK-α2) is an extremely poor substrate for Akt, and that the very low level of α2 phosphorylation obtained using Akt is not affected by an S491A mutation (Figure 1D). It had been shown previously using a bacterially expressed α2β1γ1 complex that Akt phosphorylated AMPK-α2, albeit more slowly than α1 [27] and, although the phosphorylation site(s) had not been identified, it has generally been assumed that this phosphorylation occurred at Ser^491^. That Ser^491^ is modified instead by autophosphorylation is shown by the following findings: (i) substantial phosphorylation of Ser^491^ occurred with the recombinant human α2β2γ1 complex in the absence of exogenous kinases (Figures 2A and 2B); (ii) Ser^491^ phosphorylation did not occur with an inactive (D157A mutant) complex (Figure 2B); (iii) various forms of AMPK, including the human α2β2γ1 complex, phosphorylated a synthetic peptide corresponding to the sequence around Ser^491^, but not Ser^485^ on rat α1 (Figure 2D); and (iv) Ser^491^ on AMPK-α2 became phosphorylated in response to the AMPK activator berberine in HEK-293 cells, whereas Ser^487^ on AMPK-α1 did not (Figure 4C). Although further work is required to test whether autophosphorylation of Ser^491^ down-regulates Thr^172^ phosphorylation, this is not supported by the results in Figure 2(C), where the activation of the human α2β2γ1 complex by LKB1 was not affected by a potentially phosphomimetic S491D mutation. Thus the ability of Akt-activating treatments such as insulin and IGF-1 to restrain activation of AMPK by ST loop phosphorylation is limited to complexes containing the α1 isoform.

Although Ser^487^ in the human α1β2γ1 complex also appeared to autophosphorylate to a limited extent in cell-free assays (Figure 2A), increased Ser^487^ phosphorylation did not occur when intact cells were incubated with the AMPK activator berberine (Figure 4C), suggesting that autophosphorylation of Ser^487^ is not significant in intact cells. Interestingly, the small degree of Ser^487^ autophosphorylation in cell-free assays did not increase when the α1β2γ1 complex was activated by phosphorylation at Thr^172^ by LKB1 (Figure 2B, top left), suggesting that Thr^172^ phosphorylation (unlike its effects on phosphorylation of exogenous substrates) does not enhance Ser^487^ autophosphorylation. Our results show that Ser^487^ in human α1, unlike Ser^491^ in α2, is a good substrate for Akt. Although only phosphorylated in cell-free assays at approximately 10% of the rate of Ser^9^ on GSK3-β (a canonical Akt site), in the intact cells the net phosphorylation status would also be affected by the activity of protein phosphatases acting on Ser^487^. The results using MK2206 in Figures 4 and 6 clearly confirm that Ser^487^ is phosphorylated in four distinct cell lines in an Akt-dependent manner.

In good agreement with previous results obtained with the rat α1β1γ1 complex [27], prior phosphorylation of Ser^487^ on the human α1β2γ1 complex caused a 40% reduction in subsequent phosphorylation of Thr^172^ both in active (Figure 3A) and inactive (Figure 1F) AMPK complexes, and a reduction in the activation of the active complex (Figure 3B). Although a 40% effect might appear to be quite modest, the effect appears to be larger in intact cells (Figures 4 and 6), where the activities of phosphatases acting on Ser^487^ would affect the outcome. Another explanation for the different size of the effect in cell-free assays and intact cells is that other sites in the ST loop may be phosphorylated in the intact cells, a possibility discussed further below.

By using HEK-293 cells expressing recombinant AMPK-α1, we showed not only that the effect of prior IGF-1 treatment to inhibit subsequent AMPK activation by A769662 was dependent on Akt, but also that it was dependent on phosphorylation of Ser^487^, since the effect was completely abolished in cells expressing a non-phosphorylatable S487A mutant (Figure 4). It has been reported recently that Ser^491^ on AMPK-α2 can be phosphorylated by S6K1 [42], so we considered the possibility that Ser^487^ might have been phosphorylated by a kinase downstream of Akt and mTORC1, such as S6K1, rather than directly by Akt. However, the phosphorylation of Ser^487^ was not affected by rapamycin (Figure 4D), so was not catalysed by S6K1 or any other kinase downstream of mTORC1.

Using the LKB1-null G361 cell line treated with the Ca^2+^ ionophore A23187, we also showed that phosphorylation of Thr^172^, and activation of AMPK, by CaMKKβ was antagonized by prior phosphorylation of Ser^487^. This supports results obtained in cell-free assays (Figure 3C), and shows that the effect in intact cells is independent of the upstream kinase utilized.

Surprisingly, we found that phosphorylation of Akt at the mTORC2 site, Ser^473^, was blocked by certain AMPK activators including phenformin and quercetin, although not by berberine, A769662 or A23817. Although the mechanism for this effect remains unclear, it is clearly an off-target AMPK-independent effect, because it was still observed in AMPK-knockout MEFs (Supplementary Figure S2).

To place our studies in the context of tumour cells, we also addressed whether AMPK activation was down-regulated in three PTEN-null tumour cell lines derived from human cancers. Interestingly, in two glioblastoma cell lines and a breast cancer cell line in which Akt was hyperactivated due to loss of PTEN, AMPK was rather resistant to activation and Thr^172^ phosphorylation induced by the activator A769662. However, this effect was reversed by the addition of MK2206, a selective inhibitor of Akt activation that also reduced or abolished the phosphorylation of AMPK-α1 on Ser^487^, of GSK3β on Ser^9^ and of Akt itself on Thr^308^ and Ser^473^. These effects could also be reversed in DBTRG-05MG cells by re-expressing WT PTEN, but not a phosphatase-inactive (C124S) mutant. Our results suggest that a previously unrecognized effect of PTEN loss is to reduce the potential restraint on cell growth and proliferation provided by activation of AMPK. This mechanism would also be expected to operate in tumour cells in which Akt is hyperactivated due to activating mutations in subunits of PI3K, or mutation or overexpression of upstream receptors [26]. Previous evidence suggests that this mechanism also operates in human hepatoma (Huh-7) cells infected with the hepatitis C virus [30], where PI3K is activated due to association with a non-structural protein encoded by the viral RNA [43]. In that case, expression of viral proteins was reduced by treating the infected Huh-7 cells with AMPK activators such as AICAR (5-amino-4-imidazolecarboxamide riboside) or metformin [30].

Finally, our results suggest a molecular mechanism by which prior phosphorylation at Ser^487^ inhibits subsequent phosphorylation of Thr^172^, and hence activation, by upstream kinases. In a partial α1β2γ1 complex containing rat α1, which was expressed in bacteria and where Ser^485^ was therefore most likely unphosphorylated, the ST loop from Glu^469^ to Val^524^ was not resolved, indicating that it was mobile within the crystal [11]. Our hypothesis is that the ST loop interacts with residues within the kinase domain when Ser^487^ is phosphorylated, hindering the ability of upstream kinases to gain access to Thr^172^. This hypothesis is supported by the results in Figure 7, showing that a peptide corresponding to the sequence from Arg^466^ to Asp^525^ on human α1 inhibits activation and Thr^172^ phosphorylation of an α1β2γ1 complex by LKB1, but only when phosphorylated on the residue corresponding to Ser^487^. This is an extremely specific effect, because the phosphopeptide had no effect on the activation or phosphorylation by LKB1 of BRSK2, which (with BRSK1) has the kinase domain most closely related to AMPK-α1 and AMPK-α2 within the human kinome.

The 54 residues of the ST loop in human AMPK-α1 contains 15 serine residues (including Ser^487^) and five threonine residues, most of which are conserved in α1 subunits from other vertebrates and in *C. elegans* (Supplementary Figure S1). It has been shown recently that GSK3β phosphorylates the ST loop at multiple sites, with site-directed mutagenesis suggesting that the initial phosphorylation was at Thr^481^, followed by Ser^477^ and perhaps Thr^473^ (human α1 residue numbering; in rats the equivalent residues are Thr^479^, Ser^475^ and Thr^471^). Thr^481^ phosphorylation was proposed to inhibit net Thr^172^ phosphorylation by enhancing its sensitivity to dephosphorylation [31]. With most substrates, phosphorylation by GSK3 requires ‘priming’ by another kinase, because the kinase usually phosphorylates a serine or threonine residue located four residues N-terminal to an existing phosphoamino acid [44]. In the case of AMPK it was proposed that phosphorylation of Ser^487^ on rat AMPK-α1 might promote phosphorylation of Thr^481^, although not by conventional priming because the residue spacing is not appropriate, and because phosphorylation was not affected by a GSK3β mutation that reduces phosphorylation of primed substrates [31]. If the hypothesis by Suzuki et al. [31] is correct, phosphorylation of Ser^487^ may lead to additional phosphorylation events within the ST loop. This might explain why we observed a larger effect on AMPK activation and Thr^172^ phosphorylation by modulation of Akt in intact cells than in cell-free assays (compare Figures 1 and 3 with Figures 4–6). Although GSK3β was phosphorylated at Ser^9^ in response to Akt treatment and this normally inhibits GSK3 activity [45], this inhibition does not occur with ‘unprimed’ substrates [46] as proposed for Thr^481^ [31]. Thus it is possible that phosphorylation of Ser^487^ in our intact cell experiments promoted additional phosphorylation events, such as phosphorylation of Thr^481^ and Ser^477^ by GSK3.

As an extension of this hypothesis, we propose that the side chains of three basic residues located in the αC helix of the small lobe of the kinase domain (Arg^64^, Lys^71^ and Arg^74^ in human α1) interact with multiple phosphate groups attached to the ST loop, thus anchoring the ST loop to the kinase domain and blocking access of Thr^172^ to upstream kinases. Interestingly, although at least one of these (Arg^64^ or Lys^71^) is conserved in all 12 AMPK-related kinases, none are conserved in the archetypal serine/threonine kinase domain of PKA. Consistent with our hypothesis, a human α1β2γ1 complex containing an ‘AAA’ mutation (R64A/K71A/K74A) was completely resistant to the ability of previous Akt phosphorylation to reduce the rate of Thr^172^ phosphorylation by LKB1 (Figure 7D). Also consistent with this model was our finding that prior Akt phosphorylation reduced activation by both upstream kinases (LKB1 and CaMKKβ) to very similar extents (Figure 3C). Final confirmation of this model will require structural analysis of AMPK complexes where the ST loop is present in a phosphorylated form, rather than being unphosphorylated or deleted as in existing structures [10,11].

Since AMPK activators such as AICAR or metformin can overcome the inhibitory effects of Ser^487^ phosphorylation on replication of the hepatitis C virus [30], our present results raise the exciting prospect that AMPK activators such as metformin, which are already used to treat Type 2 diabetes, might also be efficacious in treatment of tumours in which the Akt pathway is hyperactivated. It is already known from retrospective studies that treatment of diabetics with metformin is associated with a lower incidence of cancer compared with other medications [47,48], although it is not yet certain that this effect is mediated by AMPK. Our results suggest that clinical trials to test the efficacy of metformin for cancer treatment might be targeted at specific classes of tumour, such as those in which Akt is hyper-activated.

## Online data

Supplementary data
