# Supplementary material for: Phosphorylation by Akt within the ST loop of AMPK-α1 down-regulates its activation in tumour cells
Source: Biochem J. 2014 Mar 28;459(Pt 2):275–87. doi: 10.1042/BJ20131344 (PMC4052680; doi:10.1042/BJ20131344)
Supplement: Supplementary data [file bj4590275add.pdf]

BJ Signal

online data

SUPPLEMENTARY ONLINE DATA

Phosphorylation by Akt within the ST loop of AMPK-α1 down-regulates its activation in tumour cells

Simon A. HAWLEY*, Fiona A. ROSS*, Graeme J. GOWANS*, Priyanka TIBAREWAL*[1], Nicholas R. LESLIE*[1] and D. Grahame HARDIE*[2]

*Division of Cell Signalling & Immunology, College of Life Sciences, University of Dundee, Dundee DD1 5EH, Scotland, U.K.

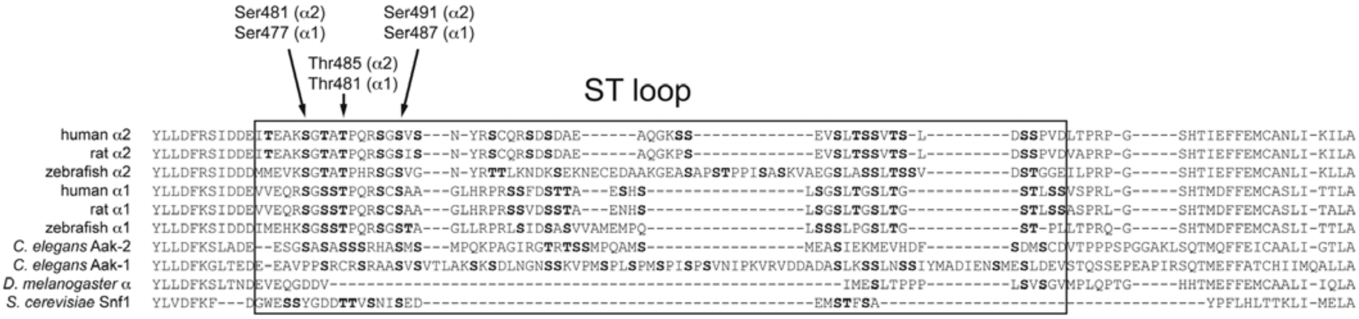

Figure S1 C-terminal sequences of selected vertebrate and non-vertebrate AMPK-α subunits, showing the location of the ST loop (boxed)

Sequences were aligned using CLC Main Workbench 6 using a 'gap open cost' of 10 and a 'gap extension cost' of 1. Note that the ST loop is well conserved between vertebrates and *C. elegans*, but poorly conserved and truncated in the sequences from insects (*D. melanogaster*) and fungi (*S. cerevisiae*). Serine and threonine residues within the ST loop are highlighted in bold font. The positions of Ser^{487}/Ser^{491}, Thr^{481}/Thr^{485} and Ser^{477}/Ser^{481} (AMPK-α1/AMPK-α2 respectively, human numbering; see the main text for details) are indicated.

[1] Present address: Institute of Biological Chemistry, Biophysics and Bioengineering, School of Engineering and Physical Sciences, Heriot Watt University, Edinburgh EH14 4AS, Scotland, U.K.

[2] To whom correspondence should be addressed (email d.g.hardie@dundee.ac.uk).

© 2014 The Author(s)
This is an Open Access article distributed under the terms of the Creative Commons Attribution Licence (CC-BY) (http://creativecommons.org/licenses/by/3.0/) which permits unrestricted use, distribution and reproduction in any medium, provided the original work is properly cited.

Biochemical Journal www.biochemj.org

A

B

C

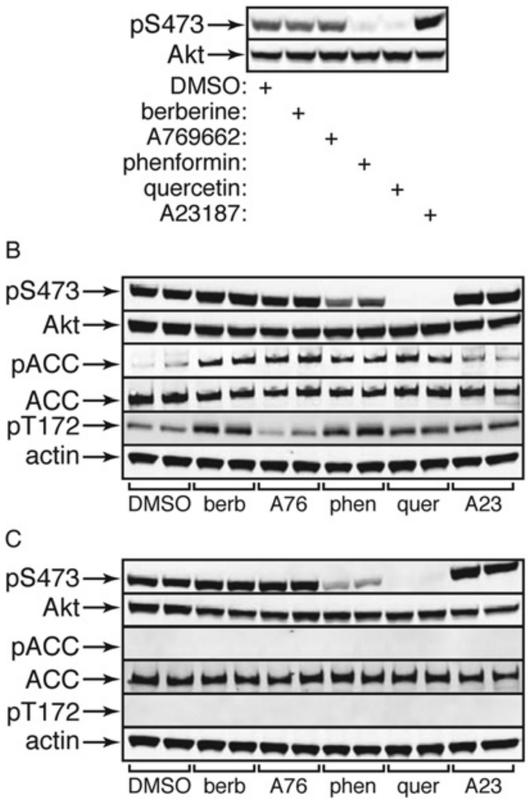

Figure S2 Some AMPK activators inhibit Akt in an AMPK-independent manner

(**A**) DBTRG-05MG cells were incubated for 60 min with vehicle (DMSO), berberine (300 μM), A769662 (300 μM), phenformin (10 mM), quercetin (300 μM) or A23187 (10 μM) and lysates were analysed by Western blotting using anti-pS473 and anti-Akt antibodies. (**B**) WT MEFs were incubated in duplicate dishes with DMSO, berberine, A769662, phenformin, quercetin or A23187 as in (**A**), and lysates analysed by Western blotting using the indicated antibodies. (**C**) As with (**B**), but using AMPK-knockout (KO) MEFs.

Received 9 October 2013/16 January 2014; accepted 27 January 2014
Published as BJ Immediate Publication 27 January 2014, doi:10.1042/BJ20131344

© 2014 The Author(s)
This is an Open Access article distributed under the terms of the Creative Commons Attribution Licence (CC-BY) (http://creativecommons.org/licenses/by/3.0/) which permits unrestricted use, distribution and reproduction in any medium, provided the original work is properly cited.
